# RT-qPCR Analysis of 15 Genes Encoding Putative Surface Proteins Involved in Adherence of *Listeria monocytogenes*

**DOI:** 10.3390/pathogens5040060

**Published:** 2016-10-01

**Authors:** Hung King Tiong, Peter M. Muriana

**Affiliations:** 1 Department of Animal Science, Monroe Street, Oklahoma State University, Stillwater, OK 74078, USA; htiong@ostatemail.okstate.edu; 2 Robert M. Kerr Food & Agricultural Products Centre, 109 FAPC Building, Monroe Street, Oklahoma State University, Stillwater, OK 74078-6055, USA

**Keywords:** *L. monocytogenes*, adherence, biofilm, expression, surface proteins

## Abstract

*L. monocytogenes* adherence to food-associated abiotic surfaces and the development of biofilms as one of the underlying reasons for the contamination of ready-to-eat products is well known. The over-expression of internalins that improves adherence has been noted in cells growing as attached cells or at elevated incubation temperatures. However, the role of other internalin-independent surface proteins as adhesins has been uncharacterized to date. Using two strains each of weakly- and strongly-adherent *L. monocytogenes* as platforms for temperature-dependent adherence assays and targeted mRNA analyses, these observations (i.e., sessile- and/or temperature-dependent gene expression) were further investigated. Microplate fluorescence assays of both surface-adherent strains exhibited significant (*P* < 0.05) adherence at higher incubation temperature (42 °C). Of the 15 genes selected for RT-qPCR, at least ten gene transcripts recovered from cells (weakly-adherent strain CW35, strongly-adherent strain 99-38) subject to various growth conditions were over expressed [planktonic/30 °C (10), sessile/30 °C (12), planktonic/42 °C (10)] compared to their internal control (16SrRNA transcripts). Of four genes overexpressed in all three conditions tested, three and one were implicated as virulence factors and unknown function, respectively. PCR analysis of six unexpressed genes revealed that CW35 possessed an altered genome. The results suggest the presence of other internalin-independent adhesins (induced by growth temperature and/or substratum) and that a group of suspect protein members are worthy of further analysis for their potential role as surface adhesins. Analysis of the molecular basis of adherence properties of isolates of *L. monocytogenes* from food-associated facilities may help identify sanitation regimens to prevent cell attachment and biofilm formation on abiotic surfaces that could play a role in reducing foodborne illness resulting from *Listeria* biofilms.

## 1. Introduction

*L. monocytogenes* is a Gram-positive, intracellular foodborne human pathogen, capable of surviving antimicrobial hurdles such as limited oxygen [1], degenerative agents associated with immunological response (phagocytosis) [2], bile salts (10%) [3], and extreme temperatures (−0.4–50 °C) [3]. The systemic disease it causes is termed listeriosis and it has a multitude of diagnostic manifestations such as miscarriage, muscle pain, stillbirth [4], meningitis [5], septicemia [2], pneumonia [6], corneal ulcers [7], fever, and gastroenteritis [8] in patients. In large outbreaks it has among the highest mortality rates (20%–25%) as compared to other foodborne pathogens reported by the Centers for Disease Control and Prevention [9]. These stress tolerant characteristics have been linked to the pathogen’s molecular defense mechanisms contributed by proteins with essential roles such as biofilm-associated protein (BapL) [10], general stress-response regulation by sigma factor B (SigB), membrane lysis by Listeriolysin O (Hly), and phospholipase during phagocytosis for cell sustainability/viability to intracellular stresses [2]. Other *Listeria* virulence factors include adhesins for attachment and invasins to gain entry into host cells (InlA, lmo0433; InlB, lmo0434; Vip, lmo0320; Ctap, lmo0135; FbpA, lmo1829; IspC; Ami, lmo2558; LapB, lmo1666; Iap 60, lmo0582) and cell-to-cell movement mediated by the polymerization of actin (ActA, lmo0204) [2]. 

Persistence of this bacterium in food products manufactured under standard sanitation protocols, especially with ready-to-eat (RTE) processed foods such as dairy products, meats, vegetables, and fish [11,12] are generally caused by cross-contamination of foods contacting *L. monocytogenes*-contaminated surfaces. Biofilm formation following initial adherence increases the cell’s resistance to elimination and removal by current sanitation regimens [13,14,15,16]. Isolates of *L. monocytogenes* from raw and processed meats and food processing facilities are capable of adhering to numerous substrate surfaces such as stainless steel, polystyrene, rubber, plastic, and glass, and different strains display different degrees of adherence [17,18]. They also demonstrated that although the weak and strongly-adherent variants adhered equally well to biotic cells, the strongly-adherent strains were more invasive as demonstrated in virulence assays in Caco-2 tissue culture and live mouse assays [19,20]. Studies by other investigators have demonstrated that adherence strength may be correlated to incubation temperature [3,21]. To date, four surface-associated adhesins, including *inl*A, *inl*B, *bap*L, and the *Staphylococcus epidermidis* *ami* homolog *atl* (lmo2558), have been characterized by different groups for attachment to abiotic surfaces [10,22,23]. However, single mutants (*inl*A, *inl*B, *bap*L, *ami*) or double deletions (*inl*A and *inl*B) did not abolish abiotic attachment completely suggesting that adherence is mediated by multiple loci or factors. 

The purpose of this study was to examine the expression of surface-associated proteins that were previously implicated as potential candidates for involvement with surface adherence based on comparative MS-LC/MS analyses of different phenotypic strains and growth conditions. Insights on attachment mechansims may provide for more effective sanitation of food processing facilities. In this study, mRNA levels of gene transcription were evaluated for 15 genes encoding cell surface proteins identified previously as potentially involved with attachment to abiotic surfaces [24]. These results are compared to those of Chen et al. [25] who evaluated two genes, *inl*A and *inl*B to establish positive correlations between gene expression and attachment strength of two adherent phenotypes of *L. monocytogenes*. Gene targets were determined based on multiple LC-MS/MS comparative analyses of surface sub-proteome extracts of adherence variants of *L. monocytogenes* (CW35/weak vs. 99-38/strong) [24]. A group of 15 genes, including a 16S rRNA reference gene [26], *inl*A [25], and 14 other target genes suggested in LC-MS/MS data were utilized for this purpose [24].

## 2. Results

### 2.1. Adherence Properties of Various Strains of L. monocytogenes

**Figure 1:**
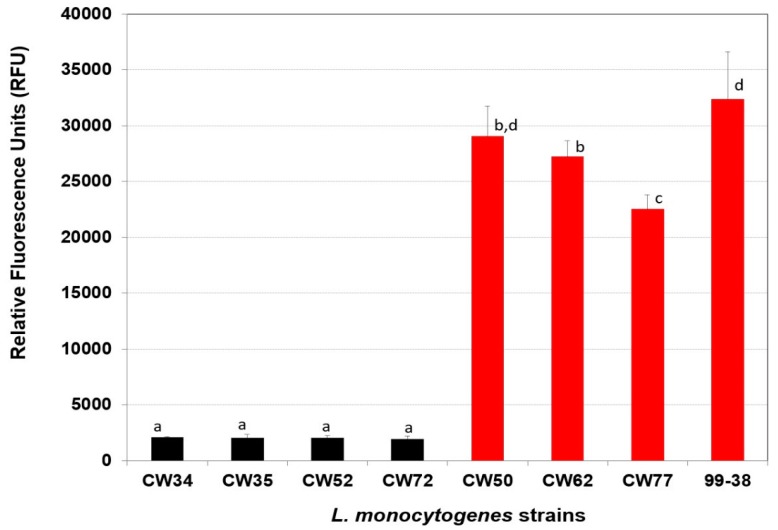
Adherence of various strains of *L. monocytogenes* using the microplate fluorescence (5,6-CFDA) adherence assay. Weakly- and strongly-adherent strains are represented by black and red bars, respectively. Data bars represent the mean of triplicate replications. Means that share the same lowercase letters are not significantly different; means with different letters are significantly different (*P* < 0.05). The error bars indicate standard deviation from the mean.

**Figure 2:**
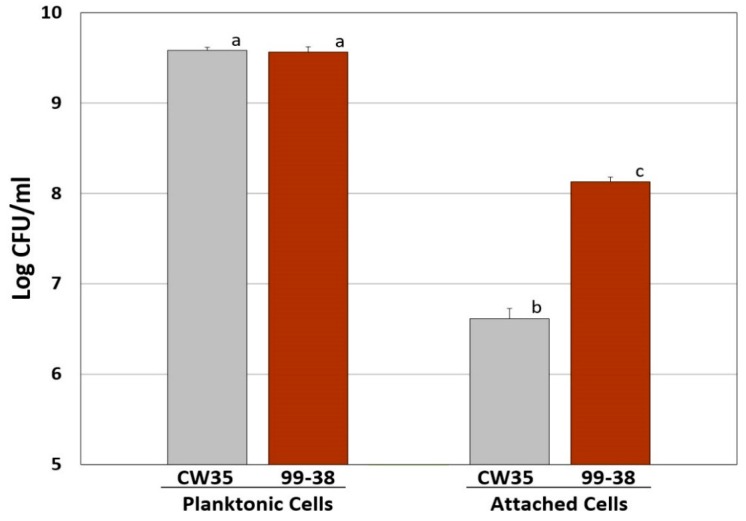
Comparison of attachment characteristics of *L. monocytogenes* CW35 (weakly-adherent) and 99-38 (strongly-adherent) in microplate wells. Enumeration of well cell cultures (left) and attached cells (right) after release by treatment with protease. All data represent the means of triplicate replications. Means with the same lowercase letters are not significantly different; means with different letters are significantly different (*P* < 0.05). Error bars indicate standard deviation from the mean. CFU, colony forming units.

In the current study, a group of 15 test genes implicated in LC-MS/MS analyses of surface sub-proteomes of *L. monocytogenes* as suspect adhesins were derived from (two each) strongly- and weakly-adherent phenotypic groups of *L. monocytogenes*; the strains used in the current study were from the same *L. monocytogenes* food isolates (i.e., CW35 and 99-38) used in a prior LC-MS/MS study [24]. Using a fluorescent microplate adherence assay [18] (Table 1) eight previously characterized strains of *L. monocytogenes* were confirmed as belonging to two distinct adherence groups of *L. monocytogenes* (Figure 1 and Figure 2). Strongly-adherent strains (CW50, CW62, CW77, 99-38) gave greater than 10-fold higher RFU signals than weakly-adherent strains (CW34, CW35, CW52, CW72) in the microplate adherence assay, agreeing with previous published findings [17,18,19].

**Figure 3:**
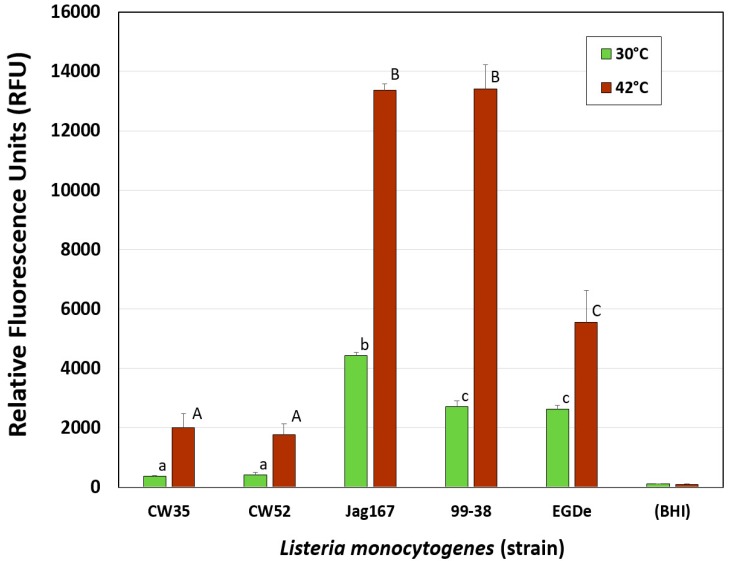
Effect of temperature (30 °C vs. 42 °C) on attachment of different adherence-variant strains of *L. monocytogenes* (strongly adherent: Jag167, 99-38, EGDe; weakly adherent: CW35, CW52) as determined by the microplate adherence assay. Uninoculated brain heart infusion (BHI) nutrient broth was tested as a control. All data represent the means of triplicate replications. Means with the same upper/lowercase letters are not significantly different; means with different upper/lower case letters are significantly different (*P* < 0.05). Error bars indicate standard deviation from the mean. RFU, relative fluorescence units.

Gorski noted that *L. monocytogenes* cells exhibited increased adherence to vegetative surfaces when higher incubation temperatures were used [3]. This observation was consistent with the results in the microplate adherence assays whereby both adherence phenotypes of *Listeria* revealed higher adherence at 42 °C incubation temperature than at 30 °C. The findings suggest that temperature may be an important factor impacting adherence of *L. monocytogenes* in food manufacturing facilities (Figure 3) [3]. 

Morange et al. [27] and Kushwaha and Muriana [19] further reported that the virulence (i.e., invasiveness) of *L. monocytogenes* was dependent upon incubation temperature and the strong adherence phenotype in *L.* *monocytogenes*, respectively, and possibly suggesting a correlation between virulence and adherence factors. In regards to food processing, higher temperatures resulting in greater levels of adherence could correlate to a greater degree of equipment surface contamination and food product contamination.

### 2.2. Differential Gene Expression of Two Adherence-Variant Strains of L. monocytogenes

**Figure 4:**
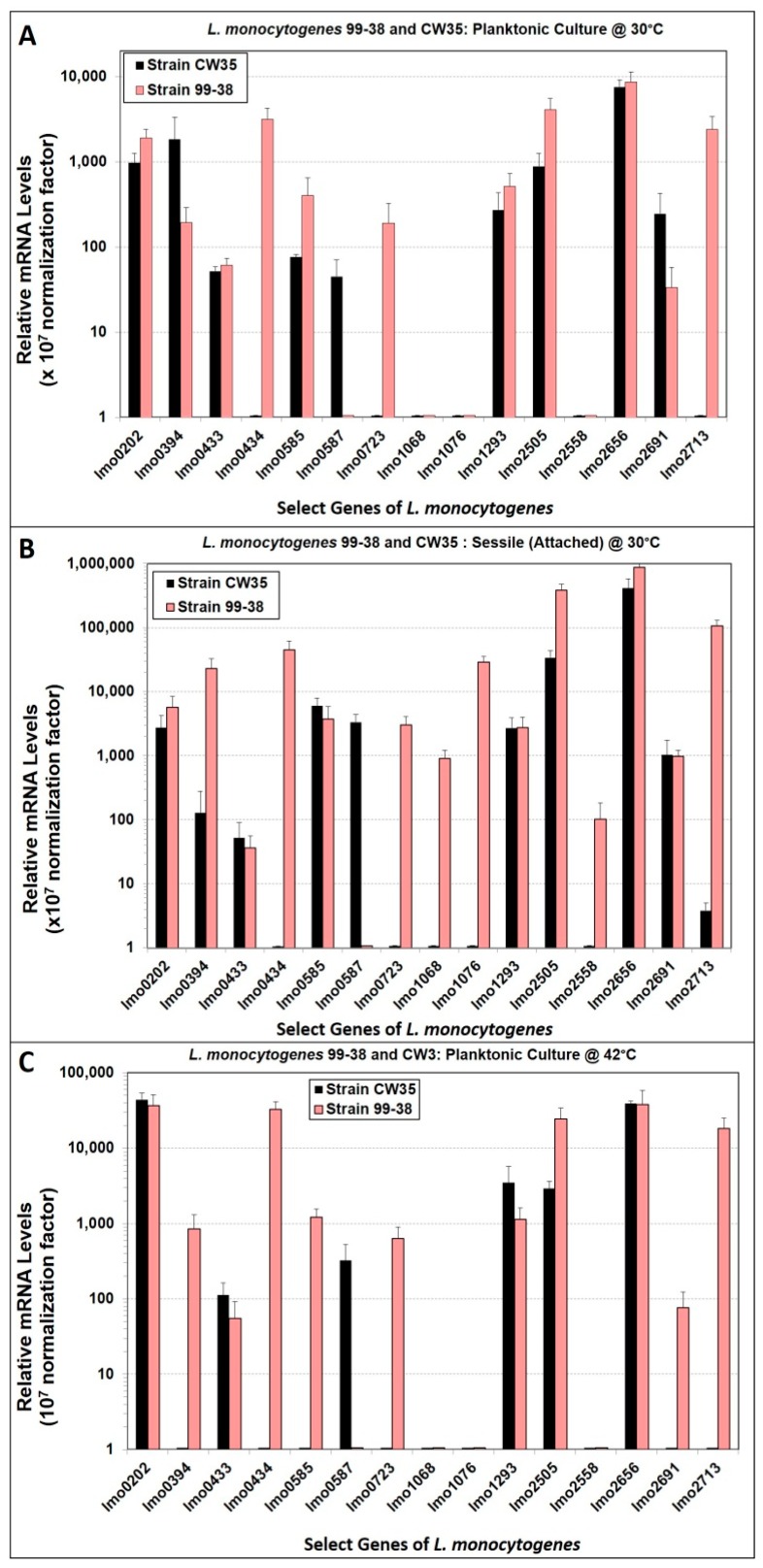
Relative transcript expression profiles of select genes from weakly-adherent (CW35) and strongly-adherent (99-38) strains of *L. monocytogenes*. Panel A, from cells recovered from planktonic growth at 30 °C. Panel B, from cells attached to glass beads during growth at 30 °C. Panel C, from planktonic cells grown at 42 °C. Expression is relative to that of the reference gene, 16S rRNA. All data bars represent the means of triplicate replications for gene expression RT-qPCR assays. Error bars indicate the standard deviation from the mean. Expression was normalized (×10^7^ factor) to eliminate negative expression levels.

A subset of transcripts of *L. monocytogenes* total RNA from weakly (CW35) and strongly (99-38) adherent phenotypes, recovered from various growth conditions such as sessile (bead attached cells) or planktonic at 42 °C or 30 °C (control), was quantitated using RT-qPCR relative to 16S rRNA. Strains of *L. monocytogenes* were initially selected based on involvement with either raw or processed meat production since both use raw meat ingredients from similar sources. Subsequent selectivity of strains was based on adherence characteristics as determined by microplate adherence assay for further analysis in the current study (Figure 1). Growth conditions were adapted from Hong et al. [29] and McGann et al. [21] for the reason that the beads used for sessile cells preparation rendered more surface area of growth than a 96-well microplate. In addition, the incubation temperature (42 °C) was the highest temperature used that rendered significant differential expression of the surface adhesins corresponding genes, *inl*A and *inl*B. Relative transcripts of both strains were obtained using a relative expression quantification method for analysis of data containing inconsistent amplification efficiencies [30] (Table 2) and the normalized data was plotted in Figure 4. Overexpressed genes (expression ≥2-fold or detected only in a single strain) were primarily attained in 99-38 cells recovered from planktonic at 30 °C (7 overexpressed in 99-38 vs. 3 in CW35) and 42 °C (7 in 99-38 vs. 3 in CW35), and from sessile cells at 30 °C (10 in 99-38 vs. 2 in CW35) (Table 3). On the other hand, four overexpressed genes (lmo0202, lmo1293, lmo2505, lmo2656) from both CW35 and 99-38 strains were detected at either elevated temperature (42 °C) or during sessile conditions (Table 4).

Nightingale [31], and Chen et al. [25] reported that truncated forms of *inl*A/B are common among *L. monocytogenes* food isolates. Similarly, we observed that CW35 chromosomal DNA possessed an altered form of *inl*A gene (3-codon deletion detected in the C-terminus) and thus producing truncated form of InlA protein in all conditions tested relative to 16S rRNA mRNA levels (data not shown).

In addition to expression variations caused by the external factors, the gene of interest might have mutations at their primer-binding regions, which could reduce the PCR amplification efficiency of that gene in comparison to other strains, and hence cause false expression levels [32]. As demonstrated in Table 5, the amplification efficiencies of each gene varied among strains tested (Table 6) and these amplification differences were corrected thereby validating our expression data [30,33,34,35].

### 2.3. PCR Amplification of Genes

**Figure 5:**
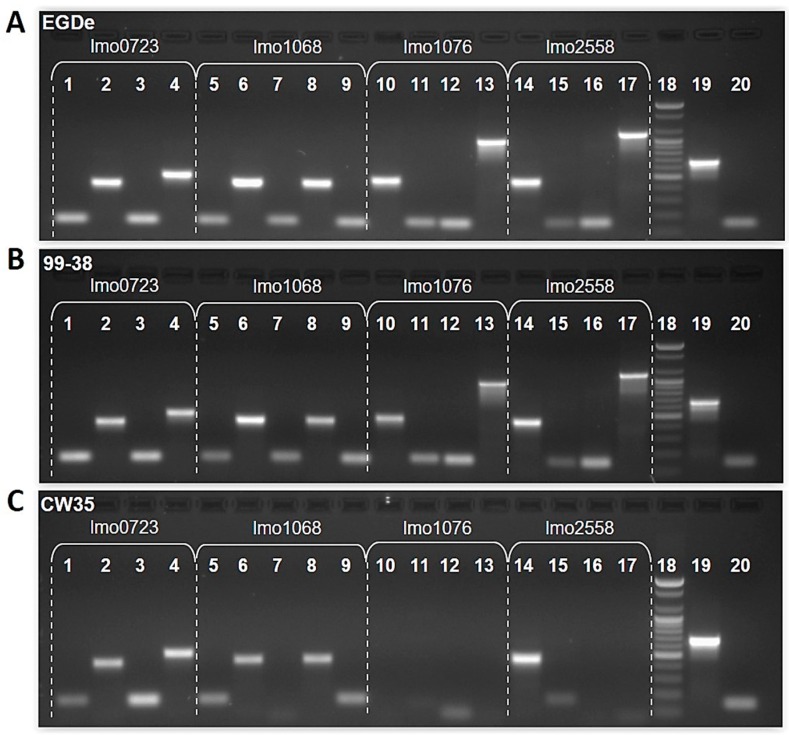
PCR products from genomic DNA of *L. monocytogenes* EDGe (Panel A), 99-38 (Panel B), and CW35 (Panel C) for PCR nucleotide evaluation of lmo0723, lmo1068, lmo1076, and lmo2558. Different gene-specific primer pairs were used for PCR amplification and subsequent agarose gel analysis of products. PCR primer combinations were based on *L. monocytogenes* type strain EGDe (Panel A) and tested on 99-38 (Panel B) and CW35 (Panel C). Gene lmo0723 : Lane 1, 0723A (148bp); 2, 0723B (416bp); 3, 0723C (150bp); 4, 0723D (505bp); lmo1068 : 5, 1068A (149bp); 6, 1068B (438bp); 7, 1068C (149bp); 8, 1068D (440bp); 9, 1068E (147bp); lmo1076 : 10, 1076A (470bp); 11, 1076B (150bp); 12, 1076C (146bp); 13, 1076D (991bp); lmo2558 : 14, 2558A (458bp); 15, 2558B (148bp); 16, 2558C (149bp); 17, 2558D (1129bp); 18, 100bp DNA ladder; 19 and 20, positive controls.

Of six genes with no detectable mRNA levels, two genes (lmo1076, lmo2558) have been reportedly absent in both *L. monocytogenes* serotypes 4a and 4b strains (Table 2, Table 3 and Table 4) [2]. PCR analysis of these genes in CW35, 99-38, and EGDe (type strain) genomes with the gene specific primers listed in Table 6, revealed normal (lmo0434, lmo0587; lmo0723, Figure 5) and altered (lmo1068, lmo1076, lmo2558; Figure 5) gene sequences based on expected amplimer size. All altered non-lethal genes were only observed in the CW35 strain. Further PCR analysis of altered genes with different primers (Table 6) suggested that the alteration was due to a deletion (lmo1076) and nucleotide alterations (lmo1068, lmo2558) (data not shown), suggesting that CW35 strain possesses altered lmo1068, lmo1076, and lmo2558 genes that may affect adherence. Thus, alterations observed with lmo1076 and lmo2558 agree with the results reported by Camejo et al. [2].

### 2.4. The Function and Virulence Information of Overexpressed Genes of L. monocytogenes

The functions of five genes (of the 15 genes examined) were determined by using Leger [36] and ListiList [37] post-genome database for *Listeria* research and functional classification tools, respectively, as their functions are currently unrevealed [2,38]. They were secreted proteins (2), ribosomal protein S12-like protein (1), methyl-accepting chemotaxis-like protein (1), and unknown protein (1) (Table 7). Of the ten remaining genes studied, seven have been experimentally characterized as virulence (6) and non-virulence (1; lmo2713) [39] factors, whereas two were Iap-like proteins (lmo0394, lmo2505), *Listeria* virulence factor [40], and one was not virulence-related as implicated in intracellular down regulation (lmo2691) [41].

## 3. Discussion

*L. monocytogenes* is often detected in food processing plants and its persistence is related to its ability to survive in environments of low temperature, pH, water activity, and the ability to form bioflms. Molecular factors involved in bacterial adherence to various abiotic surfaces has been documented by many groups [17,18,22,23,52,53,54,55,56]. Researchers have noted that *L. monocytogenes* may have multiple surface adhesins (i.e., InlA, InlB, and BapL) that participate in surface adherence [10,22,25]. It is also worth noting that the *bap*L gene is not present in all strongly adherent *L. monocytogenes* isolates [57].

In the current study, a subset of transcripts from 15 putative surface-associated adhesins overexpressed primarily in the strongly-adherent strain, *L. monocytogenes* 99-38. This could suggest characterizations of a group of potential adhesins. *Listeria* strains investigated in this study exhibited more adherence than previous reports [18,19]. This could be caused by the high temperature incubation (42 °C) of *L. monocytogenes* which could result in elevated expression of InlA and InlB surface adhesins, as noted by McGann et al. [21]. Chen et al. [22] confirmed that the adherence of *L. monocytogenes* cells on glass surfaces may be enhanced by a synergistic activity of these surface proteins and that it may be positively correlated to their expression levels [25]. Gorski et al. [3] noted that adherence of *Listeria* cells to contact surfaces was independent of flagella, and hence this gene was not analyzed in this study.

When relative gene expression levels were compared between *L. monocytogenes* CW35 and 99-38 strains, the latter strain possessed more overexpressed genes (Table 3) implicating that the strongly-adherent *L. monocytogenes* 99-38 expressed more proteins involved in surface adherence. The expression profiles of these genes (i.e., lmo0202, lmo0434, lmo1293, lmo2505, lmo2656, lmo2713) were consistent with the protein profiles attained with LC-MS/MS for surface extracts of the 99-38 *Listeria* cells attached to beads (Table 3A) [24]. Of four abundant proteins detected by LC-MS/MS in surface extracts from planktonic cells of 99-38 at 30 °C [24], only one member (lmo0723) correlated with gene expression profiles in this study. These inconsistent profiles could be partly due to the competitive (physical) detection of protein abundancy by LC-MS/MS vs. targeted gene expression studies using real-time RT-PCR as explained by others [58]. Chen et al. [22,25] observed that *L. monocytogenes* attached more strongly when the transcript levels of *inl*A/B were abundant. Surprisingly, the CW35 strain demonstrated low adherence on beads even though its relative *inl*A transcripts were similar to the control (planktonic, 30 °C) (Table 2). This observation could suggest the involvement of other adhesins [19]. 

Chen et al. [25] and Gorski et al. [3] reported that other surface adhesins are considerable and that attachment is temperature-regulated, respectively. Gene expression analysis of other select surface-associated gene products recovered from sessile or planktonic cells grown at 42 °C revealed that most of the genes tested were differentially up-regulated in one strain or another (Table 2, Table 3 and Table 4). Of four genes that appear to be upregulated in both strains when held as sessile attached cells at 42 °C (Table 2), two products (lmo0202, lmo1293) have been implicated in *Listeria* adaptation of host intracellular stresses whereas the function of lmo2656 is unknown [39,41,46]. On the other hand, two strain-specific upregulated genes (lmo2691, lmo2713) exhibited intracellular upregulations, as reported by the same groups. Camejo and et al. [2] report of *Listeria* virulence factors lmo0202 (*hly*), lmo1076 (*aut*), lmo2558 (*ami*), and lmo2691 (*mur*A) that are involved in vacuole escape, invasion, adhesion, and autolysis, respectively [2]. However, none of them have been reportedly associated with *Listeria* adhesion to abiotic surfaces. 

Chen et al. [2,22] reported that both surface-associated InlA and InlB proteins of *L. monocytogenes* promote attachment equally well to mammalian epithelial cells as well as abiotic surface adherence. Various groups have revealed that attached cells of *L. monocytogenes* to different substrate surfaces can be easily removed with protein denaturants [18,59] suggesting the proteinaceous nature of adherence factors. A mammalian epithelial cell adhesin, Ami, is known to have high levels of amino acid sequence homology to *Staphylococcus aureus* major autolysin (*atl*E) that contributes to cell adherence to polystyrene, hence suggesting that this gene may also be involved in abiotic attachment [23,48,49,50,60]. 

The genes used in this study were primarily surface-associated proteins (10), unknown (3), and cytoplasmic related (2) (Table 7). The detection of cytoplasmic-surface related proteins by LC-MS/MS analysis of surface extracts of *L. monocytogenes* suggests the involvement of moonlighting proteins that have multiple functions and locations [24,61,62,63,64,65,66]. Cytoplasmic protein lmo1293 shows considerable involvement in *Listeria* adherence as indicated by overexpressed levels of mRNA under all conditions and strains tested (Table 4). The data presented herein suggests that these genes are worthy of further investigations for potential roles as surface adhesins. Information on new adhesins may benefit food processors through improved sanitation regimens were enzyme-based sanitizers are increasingly being used to combat *Listeria* biofilms in food processing facilities to ensure RTE food products safe from contamination with *L. monocytogenes* as a public health concern.

## 4. Materials and Methods

### 4.1. L. monocytogenes Strains

Initial adherence assays were carried out with eight strains of *L. monocytogenes* (weakly adherent strains: CW34, CW35, CW52, SM3; strongly adherent strains: CW50, CW62, CW77, JAG167, 99-38). Two adherent forms of *L. monocytogenes* (CW35, 99-38) were chosen for further analysis (real-time RT-PCR). All ‘CW’ strains originated from RTE retail frankfurters whereas strains 99-38 and SM3 were isolated from retail ground beef while JAG167 was isolated from an RTE meat processing plant [9,17,19]. The bacterial strains were cultured by transferring 100 µL of thawed frozen culture suspension into 9 mL of brain heart infusion (BHI) broth (Difco; Becton-Dickinson, Franklin Lakes, NJ, USA), incubated overnight (18 to 24 h) at 30 °C and subcultured twice before experimental tests. Frozen culture stocks were prepared from 9 mL of overnight culture, centrifuged, resuspended in 2 mL of sterile BHI broth (containing 10% glycerol) and stored at −76 °C.

### 4.2. Fluorescent Microplate Adherence Assay

An adherence ability was characterized as described by Gamble and Muriana [18,19]. A consistent positive correlation between cell adhesion abilities and the viable count has been validated by many groups [17,19,20]. Briefly, each *Listeria* strain was cultured at 30 °C and diluted 5-log in fresh BHI broth, and 200 µL was transferred into designated wells of a sterile 96-well black polystyrene untreated microplates (Nunc, Roskilde, Denmark) with a clear lid, wrapped with Parafilm (Alcan Packaging, Neenah, WI, USA), and incubated at 30 °C for 24 h. Subsequently, the plate was washed three times with Tris buffer (pH 7.4, 0.05 M) in a Biotec Elx405 Magna automated plate washer (Ipswich, Suffolk, UK) to remove loosely adhered cells, and the plate washer was afterwards sanitized with 200 ppm of sodium hypochlorite (pH 6.5) after each use. The cells were subjected to another cycle of incubation in fresh BHI broth (200 µL), which was followed by washing. After the final incubation and washing, the cells were suspended in 200 μL of 5,6-carboxy-fluorescein diacetate (5,6-CFDA; Invitrogen, Carlsbad, CA, USA) fluorescent substrate solution, incubated at 30 °C for 15 min, washed (as mentioned above), and suspended with the same Tris buffer (200 μL). The plate was then read from above in a Tecan GENios fluorescent plate reader (Phoenix Research Products, Hayward, CA, USA) using a fixed signal gain of 75% (unless otherwise specified) with an excitation wavelength of 485 nm and a detection wavelength of 535 nm.

### 4.3. Extraction, Purification and Evaluation of Chromosomal DNA

Chromosomal DNA was extracted using the glass bead collision method of Coton and Coton with minor modifications [35]. Briefly, pelleted overnight cells of *L. monocytogenes* were resuspended with sterile DI water and spun down twice before subjected to bead collision in Tris buffer (10 mM, pH 8) to shear the cells and release cytosolic components. Chromosomal DNA and cell debris were spun to form supernatant and pellet, respectively. Supernatant containing DNA was aspirated into sterile Eppendorf tubes and stored at −20 °C. The quality of DNA was verified using a NanoDrop® ND-1000 spectrophotometer (Thermo Scientific, South San Francisco, CA, USA) and PCR.

### 4.4. PCR, DNA Agarose Gel Electrophoresis and Sequencing Analysis

PCR mixtures for amplification of genes were prepared according to the manufacturer’s directions for GoTaq Flexi DNA Polymerase (Promega, Madison, WI, USA). Briefly, each amplification contained 0.2 mM deoxynucleoside triphosphate mix (Fisher Scientific, Fair Lawn, NJ, USA), 1.5 mM MgCl_2_ (Promega), 1.25 U GoTaq polymerase (Promega), and 0.4 µM of primers (IDT, Coralville, IA, USA) (Table 6). The reaction conditions were programmed as follows: initial denaturation of 5 min at 95 °C, followed by 40 cycles of 1 min denaturation at 95 °C, annealing for 40 s (primer-dependent temperature; Table 1), extension for 60 s at 72 °C (Table 1), and a final extension cycle of 72 °C for 10 min before holding at 4 °C in a PTC-200 thermal cycler (MJ Research, Bio-Rad, Hercules, CA, USA). All nucleotide oligomers used in this study were generated from the specific DNA sequences of the *L. monocytogenes* type strain EGDe (NCBI) type strain by Integrated DNA technology (IDT).

PCR products were examined by agarose gel electrophoresis and purified using a Wizard SV Gel and PCR clean-up kit (Promega), and submitted to the Department of Biochemistry and Molecular Biology Recombinant DNA and Protein core facility (Oklahoma State University, Stillwater, OK) for sequence identification with a ABI 3730 DNA analyzer. 

### 4.5. Total RNA Extraction, Purification, cDNA Synthesis, Evaluation, and Real-Time Reverse Transcription PCR

#### 4.5.1. Cells Attached to Glass Beads

Strains of *L. monocytogenes* were grown in screw cap bottles containing glass beads (5 mm, 80 g) immersed in BHI broth for 18 h at 30 °C or 42°C. Each day (for 6 days), bottles of *L. monocytogenes* incubated with glass beads were decanted, washed (1× PBS) on a rotating machine (10 min per wash), and followed by another six daily cycles of incubation in fresh BHI prior to cell harvesting for total RNA extraction. At the end of incubation and washing, attached cells were harvested by gentle shaking with a reciprocating vortex shaker (MRC, Cincinnati, OH, USA) using RNAzol®RT solution and transferred into sterile Eppendorf tubes.

#### 4.5.2. Planktonic Cells

Pelleted cells of various strains of *L. monocytogenes* in sterile Eppendorf tubes were prepared from 1 mL of overnight cultures in BHI broth at 30 °C or 42 °C, and washed 3 times by suspension with 1× PBS prior to total RNA extraction.

Both washed adhered and pelleted planktonic cells were lysed by repeated pipetting in 1 mL of RNAzol®RT solution (MRC) for total RNA extraction, as instructed by manufacturer. Residual DNA was removed with gDNA wipe-out reagent included in the QIAGEN QuantiTect Reverse Transcription Kit (QIAGEN, Valencia, CA, USA) as instructed by the manufacturer. A 2.8 µL reaction mixture of genomic DNA (gDNA) wipe-out solution contained 0.15 µg of total RNA, 0.4 µL of gDNA Wipeout Buffer (7×), and RNase-free water. This reaction mixture was subsequently incubated in a water bath at 42 ºC for 2 min. The degradation of DNA was verified by PCR amplification of one of the genes to be assayed (lmo0202, *hly*) using RNA extract containing 1 µg of RNA as the potential PCR template. RNA purity and integrity were verified with UV absorbance ratio (260/280) and denaturing agarose gel (1.5%) analysis, respectively. The RNA concentration was determined with a NanoDrop ND-1000 spectrophotometer (NanoDrop Products, Wilmington, DE, USA) measured at 260 nm. RNA samples were kept at −80 °C for storage.

#### 4.5.3. Synthesis of cDNA

Synthesis of cDNA was performed using the same Qiagen kit above (QuantiTect Reverse Transcription Kit) as described by manufacturer. A 4 µL volume of cDNA synthesis buffer contained 0.2 µL Quantiscript Reverse Transcriptase, 0.8 µL of Quantiscript RT Buffer (5×), 0.2 µL of RT Primer Mix, and approximate 2.8 µL of the remaining reaction product. The reaction was then carried out at 42 °C for 30 min and finally at 95 °C for 3 min to inactivate reverse transcriptase enzyme. The formation of cDNA in the synthesis buffer was verified by PCR amplification of the *hly* gene with 0.5 µL of cDNA synthesis product as the template and agarose gel electrophoresis. The concentration was determined with a NanoDrop ND-1000 spectrophotometer measured at 260 nm. Samples of cDNA were stored at −20 °C.

#### 4.5.4. Real-Time Reverse Transcriptase Quantitative PCR

Real-time reverse transcriptase quantitative PCR (real-time RT-qPCR) of first-strand cDNA was prepared using the QuantiTect SYBR Green PCR Kit (QIAGEN) and performed in a MyiQ Real-Time PCR Detection System (Bio-Rad) as described by Xiao et al. [67]. Briefly, 10 µL of PCR reaction mixtures contained 5 µL of QuantiTect SYBR Green PCR Master Mix, 0.2 µg of the first-strand cDNA, and 0.3 µM of gene-specific primers as listed in Table 2. The real-time PCR reactions were carried out in 96-microwell plates (Axygen) for production of ~150 bp amplicons: initial denaturation at 95 °C for 10 min, and 40 cycles of denaturation at 94 °C for 15 s, annealing at 50–60 °C (based on individual PCR thermal gradient analysis) for 20 s, and extension at 72 °C for 1 min. The specificity of PCR amplifications were verified by melting curve analysis and agarose gel electrophoresis of real-time PCR products (between 50–60 °C and 95 °C). The relative expression ratios of specific genes of one strain of *L. monocytogenes* to the other were measured based on the crossing point and amplification efficiency (E) values normalized to a reference gene (16S rRNA). Expression ratio analysis (1) used the following relative quantification method, delta Ct [30,33,34] as derived from Pfaffl’s and Livak’s 2^–ΔΔCT^ method for relative quantification of gene expression to accommodate different PCR amplification efficiencies of a gene (2). PCR amplification efficiency was obtained using the formula (2) as described [30,68]. The amplification efficiency of primer sets can be found in Table 5.

Relative Expression Ratio = [(E_Ref_)^C_TTest_^/(E_Target_)^C_TTest_^]/[(E_Ref_)^C_Tcalibrator_^/(E_Target_)^C_Tcalibrator_^] (1)

Amplification Efficiency = E = {[10 ^(−1/slope)^] − 1} × 100 (2)

Identities of a subset of PCR products (i.e., lmo0202, lmo0723, lmo1293, lmo2505, lmo2656, lmo1076 amplicons) were verified by DNA sequencing at the OSU core facility.

### 4.6. Statistical Significant Measurement

Comparison studies (attachment strength or expression values) either within each strain or between strains yielded pairs of mean bars with respective standard deviation (error bars). Student’s t-test in Sigmaplot 13 was used to analyze each pair of means for determination of significant difference. Statistically significant differences between means compared were called at *P* < 0.05.

## 5. Conclusion

Adherence of *L. monocytogenes* to abiotic surfaces is a serious problem impacting sanitation in food manufacturing industry affecting persistence of the organism that may result in contamination of RTE products and human listeriosis transmitted through ingestion of contaminated foods. The ability to adhere promotes initial attachment that can lead to more fully-developed biofilms that are difficult to remove and can resist sanitization regimens. Attachment can be attributed to a group of genes encoding surface adhesins. The current relative mRNA expression study suggested new suspect adhesins based on observations with strain-specific and inducible gene expression profiles, supported by current literature on the function of closely related genes. The genes that were examined encode 5 functionally unknown proteins (lmo0723, lmo0585, lmo0587, lmo1068, lmo2656), 4 virulence proteins (lmo0202, lmo1076, lmo1293, lmo2558), 2 that were similar to other virulence proteins (i.e., Iap: lmo0394, lmo2505) and 2 that were not associated with virulence (lmo2691, lmlo2713). These additional roles as potential adhesins would further qualify them as moonlighting proteins. Knowledge of different conditions that are capable of regulating a group of adhesin genes and understanding the mechanisms leading to *Listeria* attachment, may help prevent facility contamination by manipulating physical and biological conditions. These results imply that more than one surface protein may regulate the adherence property (jointly or independently) and the role of overexpressed genes in *Listeria* adherence should be further investigated as to whether they contribute to persistent biofilms.

## Figures and Tables

**Table 1:** Strains of *L. monocytogenes* used in this study.

Strain ^a^	Serotype	Adherence phenotype ^b^	Origin of isolation	Reference
CW34	ND ^c^	Weak	RTE retail frankfurters	[9,18,19]
CW35	ND ^c^	Weak	RTE retail frankfurters	[9,18,19]
CW50	ND ^c^	Strong	RTE retail frankfurters	[9,18,19]
CW52	ND ^c^	Weak	RTE retail frankfurters	[9,18,19]
CW62	ND ^c^	Strong	RTE retail frankfurters	[9,18,19]
CW72	ND ^c^	Weak	RTE retail frankfurters	[9,18,19]
CW77	ND ^c^	Strong	RTE retail frankfurters	[9,18,19]
EGDe	1/2a	Strong	Animal (EGD derivative)	[28]
Jag167	ND ^c^	Strong	RTE meat processing facilities	[17]
99-38	ND ^c^	Strong	Retail ground beef	[18,19]

^a^ *L. monocytogenes* strains 99-38, CW and Jag were isolates from our collection; ^b^ Determined by microplate adherence assay [18]; ^c^ ND, not determined.

**Table 2:** Relative mRNA levels of 15 genes as compared to the reference gene (i.e., 16S rRNA gene).

Gene Name	*L. monocytogenes* CW35			*L. monocytogenes* 99-38		
	Bead-sessile + 30 °C ^a^	Planktonic + 30 °C ^a^	Planktonic + 42 °C ^a^	Bead-sessile + 30 °C ^a^	Planktonic + 30 °C ^a^	Planktonic + 42 °C ^a^
lmo0202	2.7E-04 (1.5E-04)	9.7E-05 (2.7E-05)	4.4E-03 (9.5E-04)	5.7E-04 (2.7E-04)	1.9E-04 (4.9E-05)	3.6E-03 (1.5E-03)
lmo0394	1.3E-05 (1.5E-05)	1.8E-04 (1.5E-04)	0.0E+00 (0.0E+00)	2.3E-03 (1.0E-03)	1.9E-05 (9.7E-06)	8.5E-05 (4.7E-05)
lmo0433	5.2E-06 (3.8E-06)	5.2E-06 (7.0E-07)	1.1E-05 (5.0E-06)	3.6E-06 (1.9E-06)	6.1E-06 (1.3E-06)	5.6E-06 (3.6E-06)
lmo0434	0.0E+00 (0.0E+00)	0.0E+00 (0.0E+00)	0.0E+00 (0.0E+00)	4.5E-03 (1.7E-03)	3.1E-04 (1.1E-04)	3.3E-03 (8.7E-04)
lmo0585	6.00E-04 (1.94E-04)	7.59E-06 (4.86E-07)	0.00E+00 (0.00E+00)	3.73E-04 (2.07E-04)	4.04E-05 (2.42E-05)	1.21E-04 (3.57E-05)
lmo0587	3.3E-04 (1.0E-04)	4.5E-06 (2.6E-06)	3.3E-05 (2.0E-05)	0.0E+00 (0.0E+00)	0.0E+00 (0.0E+00)	0.0E+00 (0.0E+00)
lmo0723	0.0E+00 (0.0E+00)	0.0E+00 (0.0E+00)	0.0E+00 (0.0E+00)	3.0E-04 (1.1E-04)	1.9E-05 (1.3E-05)	6.3E-05 (2.7E-05)
lmo1068	0.0E+00 (0.0E+00)	0.0E+00 (0.0E+00)	0.0E+00 (0.0E+00)	9.1E-05 (2.9E-05)	0.0E+00 (0.0E+00)	0.0E+00 (0.0E+00)
lmo1076	0.0E+00 (0.0E+00)	0.0E+00 (0.0E+00)	0.0E+00 (0.0E+00)	2.9E-03 (5.9E-04)	0.0E+00 (0.0E+00)	0.0E+00 (0.0E+00)
lmo1293	2.7E-04 (1.2E-04)	2.7E-05 (1.6E-05)	3.5E-04 (2.3E-04)	2.8E-04 (1.2E-04)	5.2E-05 (2.1E-05)	1.1E-04 (4.7E-05)
lmo2505	3.4E-03 (1.0E-04)	8.8E-05 (3.8E-05)	2.9E-04 (7.6E-05)	3.8E-02 (1.9E-04)	4.1E-04 (1.5E-04)	2.5E-03 (9.4E-04)
lmo2558	0.0E+00 (0.0E+00)	0.0E+00 (0.0E+00)	0.0E+00 (0.0E+00)	1.0E-05 (8.1E-06)	0.0E+00 (0.0E+00)	0.0E+00 (0.0E+00)
lmo2656	4.1E-02 (1.7E-02)	7.5E-04 (1.6E-04)	3.9E-03 (3.3E-04)	8.7E-02 (2.6E-02)	8.6E-04 (2.7E-04)	3.8E-03 (2.1E-03)
lmo2691	1.0E-04 (7.2E-05)	2.5E-05 (1.8E-05)	0.0E+00 (0.0E+00)	9.7E-05 (2.4E-05)	3.3E-06 (2.5E-06)	7.7E-06 (4.6E-06)
lmo2713	3.8E-07 (1.2E-07)	0.0E+00 (0.0E+00)	0.0E+00 (0.0E+00)	1.1E-02 (2.3E-03)	2.4E-04 (9.9E-05)	1.8E-03 (6.6E-04)

^a^ Expression data represents an average of 2 technical replicates for each of 3 biological replicates with the standard deviation of the mean given in parenthesis.

**Table 3:** Select transcriptional expression comparisons (fold-differences) of *L. monocytogenes* 99-38 and CW35 cells under different conditions.

Locus Tag	Planktonic (30 °C)		Sessile (30 °C)		Planktonic (42 °C)	
	99-38	CW35	99-38	CW35	99-38	CW35
lmo0202	1.95	--	2.1	--	--	--
lmo0394	--	9.48	176.7	--	√	--
lmo0433	--	--	--	--	--	2
lmo0434	√	--	√	--	√	--
lmo0585	5.3	--	--	1.6	√	--
lmo0587	--	√	--	√	--	√
lmo0723	√	--	√	--	√	--
lmo1068	--	--	√	--	--	--
lmo1076	--	--	√	--	--	--
lmo1293	1.9	--	--	--	--	3.1
lmo2505	4.6	--	11.3	--	8.4	--
lmo2558	--	--	√	--	--	--
lmo2656	--	--	2.1	--	--	--
lmo2691	--	7.4	--	--	√	--
lmo2713	√	--	28,232.2	--	√	--

--: Neutral fold-expression; expression not detected in both strains. √: Not determined; gene expression was not detected in the other strain.

**Table 4:** Expression fold differences of 15 genes in sessile (30 °C) or planktonic (42 °C) condition compared to their planktonic equivalent at 30 °C.

Gene Annotation	Sessile (30 °C)		Planktonic (42 °C)	
	99-38 ^a^	CW35 ^a^	99-38 ^a^	CW35 ^a^
lmo0202	3.0	2.8	19.1	45.1
lmo0394	118.1	0.1	4.4	NA
lmo0433	0.6	1.0	0.9	2.2
lmo0434	14.2	NA	10.4	NA
lmo0585	9.2	79.1	3.0	NA
lmo0587	NA	74.2	NA	7.2
lmo0723	15.9	NA	3.3	NA
lmo1068	NA	NA	NA	NA
lmo1076	NA	NA	NA	NA
lmo1293	5.4	10.0	2.2	12.8
lmo2505	94.1	38.3	6.1	3.3
lmo2558	NA	NA	NA	NA
lmo2656	101.5	54.7	4.4	5.2
lmo2691	29.2	4.2	2.3	NA
lmo2713	44.6	NA	7.6	NA

^a^ Expression fold difference; a ratio of treatment/control. NA, not available; expression levels were not detectable. Brackets, group of genes that were overexpressed in both *L. monocytogenes* 99-38 and CW35 strains when each condition of sessile and 42 °C was used, as compared to growth at 30 °C.

**Table 5:** Amplification efficiency (E) and the percent efficiency (%E) of each pair of primers (based on *L. monocytogenes* EGD-e) as used in the quantitation of RTqPCR transcripts. The CW35 and 99-38 genomic DNA was used as template DNA.

Gene	CW35 ^a^ (E)	99-38 ^a^ (E)	CW35 ^a^ (%E)	99-38 ^a^ (%E)
16S rRNA	1.8	1.8	77.2	84.3
lmo0202	1.7	1.8	72.4	81.7
lmo0394	1.7	1.6	66.5	61.2
lmo0433	1.9	2.0	88.0	99.0
lmo0434	1.6	1.5	55.8	51.7
lmo0585	1.6	1.6	64.3	57.7
lmo0587	1.7	1.9	71.4	86.6
lmo0723	NA	1.7	NA	70.2
lmo1293	1.7	1.8	65.8	82.4
lmo1068	NA	1.8	NA	77.3
lmo1076	NA	1.6	NA	59.2
lmo2505	1.7	1.6	71.4	61.4
lmo2558	NA	1.7	NA	73.2
lmo2656	1.7	1.7	72.4	69.3
lmo2691	1.7	1.9	69.2	88.4
lmo2713	1.8	1.5	78.5	52.6

**^a^** CW35, weakly-adherent phenotype; 99-38, strongly-adherent phenotype. NA, not available due to no signal (Ct).

**Table 6:** Gene-specific primers used in this study.

Gene	Primer Sequence ^a^	Amplicon Size (bp)	Reference
16S rRNA	F: CGGAGCAACGCCGCGTGTATGAAGAA R: TATTACCGCGGCTGCTGGCACGTAGTTA	146	[26,42] This RT-PCR study
lmo0202	F: ACGGAGATGCAGTGACAAATG R: TGGATAGGTTAGGCTCGAAATTG	146	This RT-PCR study
lmo0394	F: GGAAAGTTGGTTATGTTTCAGG R: AAACAGCTTGGGCCAGTAG	145	This RT-PCR study
lmo0433	F: TGTTACAAGAACCTACGGCACCAACAA R: TTGGCGCTATATTGGGCATATAAGGTGATG	145	This RT-PCR study
lmo0434	F: AACCTTTCCTTAGACCGATACG R: TTGGTAGACCGATAGCTTATTCAC	150	This RT-PCR study
lmo0585	F: TGGAACTTCAATCGTGAGTGTTG R: AGTGTTGCGCTTCCTGCTG	147	This RT-PCR study
lmo0587	F: ACAATAGCGTCCGTTGTATCTGG R: TTACTTCAGCCGTTCCACCAC	148	This RT-PCR study
lmo0723C	F: TGGTTTCGCAGTCGTAGCCGAAGAA R: GCTTCGGATTCGGAAAGACCTGTGTTCA	150	This RT-PCR study
lmo1068A	F: TTCTTGGTGGAGATGTAACAACGACGTATT R: ACTTTCTGGGTTACTCGCACTTACTTCTTT	149	This RT-PCR study
lmo1076C	F: CTAATGGTTTATGGTCTGAGGTTCCAGGT R: ACCGCCTACTTGGAATTGATAGTAAGTTCG	146	This RT-PCR study
lmo1293	F: TTAGAAGAAGGCCGTGAGATGG R: GCTTCATGTTGAATTGAGTAGCGTAG	146	This RT-PCR study
lmo2505	F: ATCACGTTCACTTACAAGACCAG R: GAAGATCAAGCAACAGCAATTC	150	This RT-PCR study
lmo2558C	F: AGCTCTAACACTCCAACGAGAAGCTACGA R: TGACGCGACTATATGCAGTGATGGCTTTG	149	This RT-PCR study
lmo2656	F: CACTATGTTCTTGTAAGTTGTGACC R: AACGTGGCGTATGTACTCG	147	This RT-PCR study
lmo2691	F: AATGCAACAAGCTCTTCTACACC R: CATGACAGATGCGTACAGGTC	150	This RT-PCR study
lmo2713	F: AAGGCACGTGAGTCAATCC R: GTAGTAGTGTTAAGTACCTCGGTTCAG	145	This RT-PCR study
1mo1076B	F: CGTTATGCAACGGACAACAC R: ACCATGCCCATCTGCTTTA	150	This PCR study
lmo1076A	F: TATGGCTGCTTTAGTCGTGCCTCA R: TGTCCGTTGCATAACGTCCCTGTA	470	This PCR study
lmo1076D	F: TATGGCTGCTTTAGTCGTGCCTCA R: ACCGCCTACTTGGAATTGATAGTAAGTTCG	991	This PCR study
lmo2558B	F: TTA GGC GGAACAACCCATAC R: AGGCAGTGATTGCTTTATCATATT C	148	This PCR study
lmo2558A	F: TTGCTTCGCGCAACAACAGGATAC R: ACTGTTCCTTTGCCATCACTGTGC	458	This PCR study
lmo2558D	F: TTGCTTCGCGCAACAACAGGATAC R: TGACGCGACTATATGCAGTGATGGCTTTG	1129	This PCR study
lmo1068C	F: TAAGTGCGAGTAACCCAGAAAG R: CCCGCCGACAGATTTACTT	149	This PCR study
lmo1068B	F: CTTGGTGGAGATGTAACAACGACG R: TGGATCTGGTACGCCTATTTGCGA	438	This PCR study
lmo1068D	F: TTCTTGGTGGAGATGTAACAACGACGTATT R: TGGATCTGGTACGCCTATTTGCGA	440	This PCR study
lmo1068E	F: CTTGGTGGAGATGTAACAACGACG R: ACTTTCTGGGTTACTCGCACTTACTTCTTT	147	This PCR study
lmo0723A	F: CGCCGTGCTAATTTCCTTATTC R:GCCCAGTTCATCTCTACCATT	148	This PCR study
lmo0723B	F: TGATGGGCGAACAAATCCAAACCC R: AACAGCAAGACGTGATTGTTCCGC	416	This PCR study
lmo0723D	F: TGATGGGCGAACAAATCCAAACCC R: GCTTCGGATTCGGAAAGACCTGTGTTCA	505	This PCR study

**^a^** F, forward; R, reverse.

**Table 7:** Functional and virulence information of 15 gene targets.

Locus Tag	Gene Name [36]	^a^ Subcellular Localization	Function	Virulence Determinant
lmo0202	*hly*	Extracellular [43]	Listeriolysin, vacuole escape [2].	Yes. Validated [2].
lmo0394	--	Extracellular^P^	*Listeria* extracellular P60 protein, Iap-like protein, reduced invasion in mutant [40].	Yes. Not validated [40].
lmo0433	*inl*A	Cell wall [44]	Internalin, promote adhesion to and invasion into host intestinal epithelial cells [2]. Promote adhesion to glass surface [22,25].	Yes. Validated [2,22,25].
lmo0434	*inl*B	Cell wall [44]	Internalin, promote adhesion to and invasion into host liver cells. Involved in placental invasion [2] and adhesion to glass surface [22,25].	Yes. Validated [2,22,25].
lmo0585	--	Unknown^LP^	Putative secreted protein [36,37].	Not studied.
lmo0587	--	Unknown^LP^	Putative secreted protein [36,37].	Not studied.
lmo0723	--	Cytoplasm^P^	Methyl-accepting chemotaxis-like protein [36,37].	Not studied.
lmo1068	--	Unknown^LP^	Unknown function [36,37].	Not studied.
lmo1076	*aut*	Cell wall [2]	Promote entry into different mammalian epithelial cell lines. Virulence factor [2,45].	Yes. Validated [45].
lmo1293	*glp*D	Cytoplasm^P^	Glycerol-3-phosphate dehydrogenase. Promote intracellular virulence [46].	Yes. Validated [46].
lmo2505	*spl*	Cell wall^L^	Peptidoglycan lytic protein P45 [47]. Iap-like protein, reduced invasion in mutant [40].	Yes. Not validated [40].
lmo2558	*ami*	Extracellular [2]	Autolytic amidase, promote adhesion to mammalian epithelial cells. Virulence factor [2,48,49,50].	Yes. Validated [48,49,50].
lmo2656	*rps*L	Cell wall^L^	Ribosomal protein S12 [36,37].	Not studied.
lmo2691	*mur*A	Cell wall^L^	Autolysin, N-acetylmuramidase, promote cell separation [51].	No. Not validated [41].
lmo2713	--	Cell wall [41]	Unknown, secreted protein with 1 GW repeat [36,37]. Internalin-like protein [41].	No. Validated[39,41].

^a^ Subcellular localization of the gene products were determined using in-silico prediction tools [Leger (L); Psort (P)] as described [38] and experiments.
